# Intragenerational social mobility and functional somatic symptoms in a northern Swedish context: analyses of diagonal reference models

**DOI:** 10.1186/s12939-016-0499-1

**Published:** 2017-01-03

**Authors:** Frida Jonsson, Miguel San Sebastian, Anne Hammarström, Per E. Gustafsson

**Affiliations:** 1 Department of Public Health and Clinical Medicine, Unit of Epidemiology and Global Health, Umeå University, Umeå, SE-901 85 Sweden; 2 Department of Public Health and Caring Sciences, Uppsala University, Uppsala, Sweden

**Keywords:** Sweden, Social mobility, Intragenerational, Social class, Life course, Diagonal reference model, Self-reported symptoms

## Abstract

**Background:**

Research indicate that social class mobility could be potentially important for health, but whether this is due to the movement itself or a result of people having been integrated in different class contexts is, to date, difficult to infer. In addition, although several theories suggest that transitions between classes in the social hierarchy can be stressful experiences, few studies have empirically examined whether such movements may have health effects, over and above the implications of “being” in these classes. In an attempt to investigate whether intragenerational social mobility is associated with functional somatic symptoms in mid-adulthood, the current study tests three partially contrasting theories.

**Method:**

The *dissociative theory* suggests that mobility in general and upward mobility in particular may be linked to psychological distress, while the *falling from grace theory* indicates that downward mobility is especially stressful. In contrast, the *acculturation theory* holds that the health implications of social mobility is not due to the movement itself but attributed to the class contexts in which people find themselves. Diagonal Reference Models were used on a sample of 924 individuals who in 1981 graduated from 9^th^ grade in the municipality of Luleå, Sweden. Social mobility was operationalized as change in occupational class between age 30 and 42 (measured in 1995 and 2007). The health outcome was functional somatic symptoms at age 42, defined as a clustering self-reported physical symptoms, palpitation and sleeping difficulties during the last 12 months.

**Results:**

Overall mobility was not associated with higher levels of functional somatic symptoms compared to being immobile (*p* = 0.653). After controlling for prior and current class, sex, parental social position, general health, civil status, education and unemployment, the association between downward mobility was borderline significant (*p* = 0.055) while upward mobility was associated with lower levels of functional somatic symptoms (*p* = 0.03).

**Conclusion:**

The current study did not find unanimous support for any of the theories. Nevertheless, it sheds light on the possibility that upward mobility may be beneficial to reduce stress-related health problems in mid-life over and above the exposure to prior and current class, while downward mobility can be of less importance for middle-age health complaints.

## Background

One of the most urgent challenges before us today is the inability to close the health gap between people at the top and in the bottom of the social hierarchy. Irrespective of whether one refers to differences by income, education, status or class, those of privilege tend to be healthier and live longer, while a disproportionately large burden of disease is concentrated amongst the most disadvantaged groups [1]. Persisting social inequalities in health and the existence of a clear gradient in both mortality and morbidity have put social mobility in the spotlight for scholars as well as policy makers, both as a potential source of the inequality [2] and as a strategy to reduce it [3].

When speaking of social mobility, a differentiation is usually made between mobility within and between generations. Contrasting to *inter*generational social mobility which refer to people’s social position relative to that of their parents, *intra*generational social mobility (which is the focus of the present study), pertain to the movements people make across the social order throughout their own adult lifetime. The existence of a hierarchical separation between strata in society is thus essential for mobility processes to occur; as pointed out by Beller and Hout “social mobility would not matter in a society in which there was no inequality” ([4], p. 20). In a society where inequalities are prominent, it has been suggested that high levels of social mobility are desirable as it would signal an equality in life-chances and opportunity [5]. In addition, if people’s ability to move were not to be constrained by factors that are beyond their control some would argue that inequalities may indeed be tragic but not unfair [2]. It is partly based on this idea that social mobility has received attention from researchers and policy-makers alike.

In sociology where the focus on social mobility is large it tends to be seen as a population level indicator of the extent to which societies distributes opportunity justly [6]. Similarly, in social epidemiological research it is usually viewed as a process that constrains social inequalities in health since the health of mobile people tends to fall in between the class that they leave and the one which they join [7–9]. Nevertheless, although theories exist, comparatively little emphasis have been placed on empirically examining the health implications of social mobility at the individual level [10].

### Theories on intragenerational social mobility and health

Three partially contrasting although not mutually exclusive explanations as to the health effects of intragenerational social class mobility (henceforth referred to as social mobility) can be found in the literature [11]. For clarity and to contrast with the empirical hypothesizes which we aim to test, we will refer to the three explanations below as theories.

First, *the dissociative theory*, which originates from the work of Sorokin [12], suggests that mobility in general and upward mobility in particular are stressful experiences with implications in their own right. By forcing people to leave the milieu in which they feel most comfortable and thus contributing to feelings of exclusion, loneliness and isolation, mobility is by Sorokin ([12], p. 522–523) seen as a source for psychological strain and distress. The dissociative theory was partly a response to the large amount of people who saw themselves moving upward in the social hierachy post World War II [13]. As a result, although this theory is centered around the belief that any type of class transition would be demanding, Sorokin put a lot of emphasis on negative and life changing implications of upward mobility ([12], p. 508–510).

The second principal idea, *falling from grace*, emphasizes the direction of mobility by claiming that it is primarily downward movements that are harmful [11]. Downward social mobility may be indicated by a vertical change in occupation, e.g. moving from a professional job with good pay and high status to “merely” a white collar, or even a manual position [14]. Falling from grace, as described by Newman [15], thus signifies this experience, emphasizing how people who move downwards ‘had it and then they lost it’. According to her, when downward mobility is involuntary, people are often stranded between two personas, forced out of the former comfortable, fulfilling and autonomous life, and simultaneously unable to accept the new lower status identity. They tend to be subject to self-blame, anger and distress, trying desperately to hold on to a prior way of life. Similar to the dissociative theory, falling from grace suggests that mobility is a negative experience in itself with effects that can neither be attributed to nor alleviated by the new (lower) status position. Rather, this theory states that downward mobility creates enduring effects in terms of insecurity, powerlessness and resentment ([15], p. 83–90). Conditions which can be expected to have negative consequences for health.

Third and last is the *acculturation theory,* which stresses processes of resocialization. In contrast to the two above, this theory [16] emphasizes human’s ability to absorb and interact with our surroundings, thus claiming that mobile individuals have little problems maneuvering class transitions [11]. Rather than being influenced by the movement itself, this theory posits that mobile people’s health is primarily a result of being in different social contexts. However, because these individuals are believed to be adaptive (a neccessity to reasure acceptance and integration in their new environment), their health is assumed to be more strongly affected by the conditions in the class which they join than by that of the class which they leave [16, p. 294].

### Examining the health impact of intragenerational social mobility

In contemporary research the association between intragenerational social mobility and health is broadly examined through two different analytical approaches. The first is based on comparing health outcomes between groups generated by a priori or data driven development of discrete intragenerational mobility trajectories (e.g. stable high/low and upward/downward movements). Studies using these methodologies have for example found that mobile people have a higher risk of all-cause mortality compared to non-mobile individuals [17] or to the stable in the highest class [18, 19]. Similarly, a downwardly mobile trajectory has been found to be more detrimental for mental health than being stable [20], and improvements in occupational prestige across mid-life has been linked to a more favorable health development [21]. Altogether, these studies point to mobility being potentially important for health. Whether this is due to the movement itself or a result of people having faced different social norms and expected behaviors in the alternating class contexts is, however, difficult to infer.

Corresponding to Sorokin’s idea that mobility is a stressful experience, the second approach complements the first by attempting to disentangle and examine the health impact of the movement per se. In order to do this, the effect of *moving between* two classes has to be separated from those of *belonging to* them over time, something which studies using the above-mentioned trajectory methods cannot do [22]. This challenge arises because at a given point in time, a class will logically incorporate both those that have been permanently residing there as well as those that have entered it through mobility. The fact you are never without a class, but either have or have not experienced mobility, is the core of this problem and the issue that needs to be resolved in order to capture an effect of change. To date, by being the only method which allows for a differentiation between mobile and non-mobile individuals within each strata [23], Sobel’s Diagonal Reference Model (DRM) [24] is the most appropriate method to capture the effect of mobility. The utilization of DRM is fairly uncommon in research overall, with Houle [11] being one of the few who have applied it with regard to health. In his study on white men born in the late 1930’s and who graduated from high school in Wisconsin (US) in 1957, the author examined whether intragenerational social mobility was associated with psychological distress. His results did not favor either the dissociative nor falling from grace theories, but indicated the health of mobile people may be the result of an acculturation processes to the current class [11].

### A life course perspective to social mobility and health

The participants of the present study were born in Luleå, a middle sized industrial town in the north of Sweden in 1965 [25]. They came to grow up in a time with occupational instability, facing relatively high levels of youth unemployment around the time of labor market entry in the mid to late 1980s, and then a subsequent economic recession in the early 1990s. For the present cohort, class was measured in 1995 and 2007, with these people having reached age 30 and 42, respectively. At the time we start assessing their mobility, Sweden was recovering from a severe economic crisis [26]. The years to follow were, however, characterized by a steady increase in, for example gross domestic product (GDP), employment rates and disposable incomes which means that during the study period Sweden overall was fairly prosperous [27, 28].

From a life course perspective, the highest degree of mobility often come about early in the career. Adolescence and young adulthood are periods of transition with mobility being common but where a class position may be difficult to establish as ongoing military service or post-secondary education usually keep people from entering the labor market. For those who do work, however, their occupation is generally not permanent and if people are to reach high up in the social hierarchy, it is most often not until mid-adulthood that they do so. As indicated by both life course epidemiological and intergenerational social mobility research, people’s social origin is still often strongly predictive of later social positions [4] as well as of health [29].

People usually reach “occupational maturity” at some point between age 30 and 40 years [30], but for cohorts such as ours, the outset of their careers may have made occupational stability occur somewhat later than what is normally the case [31]. In addition, Sweden is a comparably fluid country [30] where generous unemployment benefits and universal access to public childcare facilities may support class movements in mid-life [6, 32]. Mobility within our sample could therefore be potentially high, although it is possible that these circumstances also allows for class transitions to be a more normative and less harmful experience. Nevertheless, the health impact of social mobility is examined at a life period during which people are often in the midst of handling multiple roles, demands and expectations, circumstances which may make them particularly sensitive to unexpected life changes [33]. Consequently, although resilience and plasticity have also been put forward as characteristics of people in mid-adulthood [34] we believe that in the context of our study mobility in itself could be important for health.

Within the present study intragenerational social mobility is examined with regard to self-reported functional somatic symptoms (FSS), which pertain to a clustering of physical complaints in the absence of an underlying organic disease [35]. Exposure to chronic stress and sustained states of negative affect is thought to increase the risk of FSS [36] and rising stress levels have indeed been shown to precede an increase in FSS [37]. Since Sorokin [12] theorized that class transitions could be linked to health via stress and strain, FSS may be a suitable health outcome to study in relation with social mobility.

### Aim and hypothesizes

The purpose of the present study is to examine whether intragenerational social mobility in mid-life is associated with higher levels of FSS, above and beyond an impact of prior and current class. Based on the dissociative, falling from grace and acculturation theories we formulated four hypothesizes which are then tested using Diagonal Reference Models – a method which is suitable because it allows us to compare the health of people that reported being in the same class between two time points (non-mobile) to the health of those who experienced a change in social class (mobile).

First, as the dissociative theory suggests that any type of mobility may be a source for distress we hypothesize that:

**Hypothesis 1**. Mobile individuals report higher levels of FSS than do non-mobile individuals.

Second and more precisely, the dissociative theory posits that upward mobility could be particularly stressful, as to why we also hypothesize that:

**Hypothesis 2**. Upwardly mobile individuals report higher levels of FSS than do non-mobile individuals.

Third, falling from grace proposes that it is primarily downward mobility may result in chronic stress and strain, we therefore hypothesize that:

**Hypothesis 3**. Downwardly mobile individuals report higher levels of FSS than do non-mobile individuals.

Fourth, distinct from and challenging both the dissociative and falling from grace theories, the acculturation theory suggests that the health of mobile people is primarily the result of being in different strata and that current class is particularly influential. Consequently, our last hypothesis is that:

**Hypothesis 4**. Current class is more important for FSS than prior class in both upwardly and downwardly mobile individuals.

## Method

### Data

For the present report we use prospective self-administrated survey data collected across 26 years through the Northern Swedish Cohort. This cohort include all students who in 1981 attended 9^th^ grade of compulsory school in the municipality of Luleå. Luleå is located in the northern part of Sweden, and have for the study period been seen as demographically comparable to Sweden as a whole [25]. Besides the initial survey carried out in 1981, four subsequent follow up questionnaires have been answered by the participants, in 1983, 1986, 1995 and 2007. Out of the initial 1071 students, 94.3% participated across the entire period (*n* = 1010). Within this study we use data from 1981, 1995 and 2007 years surveys, when the respondents were aged 16, 30 and 42, respectively.

### Measures

#### Functional somatic symptoms

Functional somatic symptoms at age 42 was operationalized by summarizing three survey questions covering ten different symptoms (cardiopulmonary/autonomic, gastrointestinal, musculoskeletal and general symptoms) occurring during the last 12 months. The first: *‘Do you have (or have you during the last 12 months) had any of the following symptoms: headache or migraine; other stomachache; nausea; backache, hip pain or sciatica; fatigue; breathlessness; dizziness; overstrain’*, covered eights symptoms with the response options ‘No’ (0), ‘Yes, light’ (1) and ‘Yes, severe’ (2). The second, pertaining to palpitation, was collected with the question: *‘How often have you had nervous problems during the past 12 months’*, with frequency of the specific symptom indicated as ‘Never’ (0), ‘Sometimes’ (1) and ‘Always’ (2). The third aimed to capture sleeplessness through the question: *‘Have you had sleeping difficulties during the past 12 months’*, the response options being ‘Never’ (0), ‘Sometimes’ (1) and ‘Often’/‘Always’ (2). The variable ranged from 0 to 18 for women and 0 to 15 for men with higher values indicating more somatic problems. Cronbach’s alpha was 0.78, and the measure has displayed acceptable factorial invariance as well as internal consistency over time [38].

#### Class and social mobility

Using the participants self-reported occupation at age 30 (1995) and 42 (2007), our operationalizations are seen as approximations of their “prior” and “current” social class across the adult life course. The occupations were originally coded according to the socioeconomic classification system (SEI) of Statistics Sweden [39] but the high resemblance with and the flexibility of the Erikson–Goldthorpe–Portocarero (EGP) class scheme [40] enabled us to operationalize two identical class variables according to the sevenfold version of this class schema [41, 42]. Although there are some differences between SEI and EGP (see e.g. [43]) the clustering of occupations according to type of employment and terms of work are similar. As a result, in a hierarchical order starting with the highest strata we had SEI category 56, 57 and 60 be the service class (I + II). SEI 46 represented the class of routine non-manual employees (III) while SEI 79 and 89 constituted the petty bourgeoisie (IVa-c). The assistant non-manual class (V) was made up of SEI 33 and 36 while SEI 21 and 22 represented the class of skilled manual employees (VI) and SEI 11 and 12 the unskilled manual class (VIIa).

From these two measures we then operationalized four social mobility variables aimed at capturing changes in class between 1995 and 2007. First, a binary variable contrasting between non-mobile (0) and mobile (1) individuals was created. Second, to account for the direction of mobility, three dummy variables were generated. The first aimed to capture upward mobility by separating upwardly mobile individuals from those experiencing downward or no mobility (1 = if upwardly mobile; 0 = other)*.* Similarly, the second dummy variable differentiated downwardly mobile people from upwardly and immobile (1 = if downwardly mobile; 0 = other). The third dummy variable contrasted between people who had not experiences any mobility from those being upwardly and downwardly mobile (1 = if non-mobile; 0 = other).

#### Control variables

The analysis also include a set of additional control variables that may be associated with both mobility and FSS. We adjust for sex and in accordance with the life course approach, also for the social group [44] of the parents when the participants were 16 years, where having two parents in manual work was defined as low class (1) while having at least one parent in non-manual or self-employed occupation indicated high class (0). To account for the possibility of health selection, rather than including earlier measures of FSS which would have rendered a large amount of non-response drop-out, we include a measure of self-rated health at age 30 (0 = good; 1 = poor or fair). We also adjust for civil status (1 = living alone) and labor market detachment (unemployment or disability pension) at age 30 (1 = yes) as well as the participant’s highest level of education (1 = less than post-secondary), measured concurrently with mobility at age 30.

To see whether this approach was too restrictive, a sensitivity analysis allowing for a more generous inclusion of covariates was run, additionally including also FSS at ages 16, 21 and 30, stressful life events at age 30 and residential mobility between 30 and 42. Besides strengthening the association between downward mobility and FSS at age 42, the main results did not change but since it reduced the sample size with ≈ 20% and introduced a non-response selection bias, the above covariates were retained.

### Analytical strategy

Diagonal Reference Models (DRMs) [45] were used for the primary analyses. The method enables the effects of moving between two classes to be examined by allowing for a proper adjustment of prior and current class. In order to capture a health effect of mobility in itself, controlling for the class variables or assess their interaction will not suffice, because in each class variable is not possible to differentiate between those who have and those who have not experienced mobility. This means that some of the mobility effects will be incorporated in the class variance ([46], p. 328–332) and as a result, the effects of moving between two classes cannot be separated from those of belonging to these classes over time.

With DRMs this problem is managed by not only redefining the class variables, but re-estimating them. The DRMs in our study will use the *estimated* mean FSS levels, not the sample mean ([24], p. 899), for people who have remained in the same strata between 1995 and 2007 to parameterize the class variables. This is seen as a plausible strategy because the characteristics of those who are permanently residing in a particular class, i.e. the immobile and stabile members, are believed to determine the attributes of that class [47]. Given the available data, this approach allows for each class variable to include only people that have not experienced any mobility. Using DRMs we are therefore able to test hypotheses 1 through 3 by allowing for the FSS levels of mobile people to be compared to the levels of FSS for non-mobile individuals in their prior and current class. In addition, the DRMs offer ‘class weights’, which reflect the proportion of FSS variance explained by prior and current class. With these we can assess the acculturation theory by observing which of these seem to be more important for FSS in mobile individuals.

The simple DRM (without mobility effects and covariates) in eq. 1 is the basis for all our analyses. Here Y_ijk_ is the level of FSS for a mobile individual in the off-diagonal cell *ij* which has *k* observations, μ_ii_ and μ_jj_ is the estimated average FSS for the immobile in the prior and current diagonal cell, *q* and (1 – *q*) are prior and current class weights and ε is an error term.

1 $$ {Y}_{ijk}=q{\mu}_{ii}+\left(1-q\right){\mu}_{jj}+{\varepsilon}_{ijk} $$

As displayed, in all our models the effects of class are adjusted for through the inclusion of the μ_ii_ and μ_jj_ parameters (estimates not reported in the results section) – a necessity for the hypotheses to be tested. To apply the DRMs we used the Diagonal Reference Function (DREF) in the General Nonlinear Models (GNM) package in R [48]. By using maximum likelihood estimation, a series of Poisson regression models were fitted, including a non-linear term which allows the variance of FSS for mobile people to depend on μ_ii_ and μ_jj_. The Akaike Information Criterion (AIC) [49] was used to asses model fit.

The analytical setups are as follows; in model 1 we test hypothesis 1 through the binary mobility variable. In model 2, we instead include the two dummy variables capturing upward and downward mobility. The third dummy variable, representing immobility, is excluded throughout the analyses allowing for this group to be the primary reference category. This enables us to test hypothesizes 2 and 3, by examining whether upwardly and/or downwardly mobile people report higher levels of FSS than non-mobile individuals. Our fourth hypothesis is examined through the class weights throughout model 1–3.

We also examined whether missing data could be a source for selection bias by having all variables predict a binary “missingness variable” (1 = missing) through a series of simple logistic regression models. The analysis did not indicate a systematic drop out related to variables in the models. For missing data on the class variable in 1995, the participants previously reported class (in 1986) was manually imputed, reducing the number of missing’s on this variable from 71 to 19. As such, complete case analyses were performed on a sample of 924 individuals.

## Results

### Descriptive statistics

Descriptive statistics of all variables in the models are presented in Table 1. In both 1995 and 2007, the petty bourgeoisie constituted the smallest class, representing 3.6 and 9% of the sample, respectively. With regard to prior class, the proportions in the different strata were fairly evenly distributed. The size of the strata in 2007 indicates that some degree of upward mobility was taking place; a smaller proportion of people reported a job which placed them in the two working class categories (16.3 and 18.4% in 2007 vs. 20.5 and 23.6% in 1995) while more people seemed to have attained a routine non-manual occupation (27.6 compared to 20.1%). As expected, across this twelve year period of mid-life, a fairly high proportion was subject to some form of mobility between 1995 and 2007 (51%), with a larger amount experiencing upward (31%) than downward (20%) mobility.

**Table 1:** Descriptive statistics for all variables and covariates in the models; mean (standard deviation) and *N* (proportions, %) in the full sample (*n* = 924)

Variables	Estimate
Functional somatic symptoms (FSS) age 42	4.21 (3.261)
Prior social class (1995)	
I + II (Service Class)	136 (14.7%)
III (Routine Non-manual)	186 (20.1%)
IVa-c (Petty Bourgeoisie)	33 (3.6%)
V (Assistant Non-manual)	162 (17.5%)
VI (Skilled Manual)	189 (20.5%)
VIIa (Unskilled Manual)	218 (23.6%)
Current social class (2007)	
I + II (Service Class)	137 (14.8%)
III (Routine Non-manual)	255 (27.6%)
IVa-c (Petty Bourgeoisie)	83 (9.0%)
V (Assistant Non-manual)	128 (13.9%)
VI (Skilled Manual)	151 (16.3%)
VIIa (Unskilled Manual)	170 (18.4%)
Social mobility	
Mobility (1 = mobile)	474 (51%)
Upwardly mobile	286 (31%)
Downwardly mobile	188 (20%)
Additional controls	
Sex (1 = men)	482 (52%)
Parental social position 1981 (1 = low)	346 (37%)
Self-rated health 1995 (1 = poor or fair)	212 (23%)
Civil status in 1995 (1 = alone)	220 (24%)
Unemployed in 1995 (1 = yes)	117 (13%)
Highest level of education (0 = post-secondary)	596 (65%)

Table 2 presents the mean levels of FSS at age 42 for mobile (off the diagonal) and immobile (in the diagonal,) individuals. For people who have stayed in the same class between 1995 and 2007, the diagonal cell display that the petty bourgeoisie report levels of FSS (*M* = 3.16) that are numerically comparable to those of the service class (*M* = 3.14). Apart from this, there seemed to be a descending trend along social class, with unskilled manual workers reporting the highest symptoms (*M* = 5.01). Concerning upwardly and downwardly mobile individuals, the descriptive statistics in Table 2 do not provide any readily apparent indications on whether or how mobility could potentially be associated with FSS. As such, we turn directly to the DRM analyses.

**Table 2:** Mean functional somatic symptoms (cell size, N) at age 42 by prior (1995) and current (2007) social class (*N* = 924)

Social class 1995	Social class 2007						
	I + II	III	IVa-c	V	VI	VIIa	Row means
I + II	3.14 (66)	4.25 (53)	2.75 (12)	1.60 (5)	-	-	3.48 (136)
III	4.11 (28)	4.05 (111)	4.64 (11)	3.53 (19)	4.43 (7)	3.20 (10)	4.01 (186)
IVa-c	2.67 (3)	-	3.16 (19)	2.25 (4)	2.80 (5)	1.50 (2)	2.85 (33)
V	5.29 (21)	3.54 (35)	4.22 (9)	4.44 (63)	6.67 (9)	4.56 (25)	4.49 (162)
VI	4.08 (12)	3.71 (28)	3.29 (21)	4.56 (18)	4.51 (84)	5.27 (26)	4.34 (189)
VIIa	3.29 (7)	4.43 (28)	4.73 (11)	4.37 (19)	4.52 (46)	5.01 (107)	4.71 (218)
Column means	3.74 (137)	4.03 (255)	3.65 (83)	4.13 (128)	4.58 (151)	4.84 (170)	*N =* 924

### Testing hypotheses of social mobility and health

Table 3 present the results from the Diagonal Reference Models (*n* = 924). In model 1, the insignificant parameter estimate (*p* = 0.653) indicates that being mobile overall is not associated with higher levels of FSS (hypothesis 1). The direction of mobility is therefore assessed in model 2 by the parameter capturing upward mobility (hypothesis 2). The results from this model suggests that, compared to being immobile, upward movements are associated with lower levels of FSS (*B* = −0.11, *p* = 0.006), even after adjusting for prior and current class. The parameter estimate for downward mobility in model 2 (corresponding to hypothesis 3) is, on the other hand, 0.08 and insignificant (*p* = 0.08). Model 3 adds the covariates and indicates that neither low origin social position (measured at age 16), unemployment at age 30 nor less than post-secondary schooling are associated to higher levels of FSS, while being a woman as well as reporting poor/fair self-rated health and living alone at age 30 are. After controlling for these variables a significant association between upward mobility and FSS persists, although becoming slightly attenuated (*B* = −0.08, *p* = 0.03). In addition, with the inclusion of covariates in model 3 the association between downward mobility and FSS grew slightly stronger (*B* = 0.09) but remains borderline significant (*p* = 0.055).

**Table 3:** Estimates (B) and 95% confidence intervals in parentheses from Diagonal Reference Models predicting functional somatic symptoms at age 42, *n* = 924

	Model 1	Model 2		Model 3	
Mobility (0 = immobile)	−0.015 (−0.08, 0.05)				
Immobile		1		1	
Upwardly mobile		−0.11 (−0.18, −0.03)		−0.08 (−0.16, −0.008)	
Downwardly mobile		0.08 (−0.008, 0.16)		0.09 (−0.002, 0.18)	
Sex				−0.24 (−0.31, −0.17)	
Parental social position				0.03 (−0.04, 0.09)	
Self-rated health 1995				0.37 (0.30, 0.44)	
Civil status in 1995				0.16 (0.09, 0.24)	
Unemployed in 1995				0.08 (−0.01, 0.17)	
Level of education				0.0006 (−0.08, 0.08)	
AIC	5034	5025		4848	
Social class weights	Mobile	Downwardly	Upwardly	Downwardly	Upwardly
Prior class (*q*)	0.75 (0.11)	1.00	0.56 (0.15)	0.69 (0.30)	0.65 (0.22)
Current class (1 – *q*)	0.25 (0.11)	0.00	0.44 (0.15)	0.31 (0.30)	0.35 (0.22)

Concerning the fourth hypothesis, the class weights in our three models indicate that for both upwardly and downwardly mobile people, prior class seems to be somewhat more important for FSS than current class. The class weights in model 3 suggest that 65% of the joint variance explained by prior and current class is attributable to prior and 35% to current class. For the downwardly mobile, the results in model 3 are similar, with prior class contributing with 69% of the joint variance explained and current class 31%.

## Discussion

The purpose of the present study was to examine whether class transitions across a twelve year period during mid-life, i.e. intragenerational social mobility, have health implications, over and above the effect of prior and current class. Following in the footsteps of Houle [11] the dissociative, falling from grace and acculturation theories were empirically analyzed using a novel method. Taken together, the study did not find unanimous support for any of the four hypotheses tested. Compared to immobility, neither overall mobility (hypothesis 1) nor upward movements (hypothesis 2) predicted higher FSS levels, thus contradicting Sorokin’s dissociative theory. Instead, after controlling for potential confounders as well as prior and current class, upward mobility was associated with lower levels of FSS. In contrast also to falling from grace (hypothesis 3), downward mobility was not strongly related to FSS at age 42. In addition, since prior class seemed to be somewhat more important for FSS than current class for both upwardly and downwardly mobile people, neither was the acculturation theory supported (hypothesis 4).

Sorokin’s [12] elaborations on how changes between class contexts should not be easily coped with but rather a very stressful experience do not seem to fit in the context of the present study. The reasons as to why mobility overall was found to have limited effects might be because up- and downward movements are, in fact, different experiences that should perhaps not be clustered together. Although Sorokin also emphasized the negative consequences of upward movements, conversely, it seemed as if people born in Northern Sweden in 1965 might experience better health as a result of upward mobility. Even after controlling for potential confounders such class of origin, which has known implications for both later health [50] and adult social positions [4], this finding persisted. Maybe the result is due to an improvement in the absolute material standard of living, or perhaps the reasons are more psychological and linked to a strengthened self-image. Goldthorpe found some support for the latter notion when British middle-aged men were asked to describe their mobility experience: “(…) in accounting for the work-life advancements of which they were so overwhelming aware, they clearly wished to represent this as being primarily their own achievements” ([51], p. 234). In contrast to this idea, however, neither Hadjar and Samuel [52] nor Marshall and Firth [53] has found an upward trajectory to be associated with higher life satisfaction/well-being.

According to Newman [15], downward mobility should be a source for negative affect and psychological distress. However, when examined with regard to self-reported functional somatic symptoms in this study, the implications of such movements, over and above the importance of prior and current class, appeared small. Notwithstanding that downward mobility is probably a disruptive life event in some way, especially if involuntary, Hout and DiPrete [6] suggest that its negative effects may be partially alleviated. Generous and universal social security systems could potentially remove some desperation when downward mobility is at risk (e.g. in times of downsizing, job insecurities/displacements and unemployment) and consequently function as a buffer. As such, in the context of the present study it is possible that downward mobility simply does not act on health as described by Newman [15].

Lastly, the acculturation theory claims that there are no effects of moving per se but rather that the health of mobile people is a result of being in different class contexts [16]. In the present study we found that upward movements might be beneficial, but also that both prior and current class seem to take part in shaping the health of mobile people. As such, at a general level, our results seem to favor resocialization as a partial explanation as to how social mobility may impact on health, but more specifically, the findings were not in the expected direction. While acculturation holds that current class is to have a stronger impact than prior, the class weights in our study did not support such a notion. Instead, similar to the studies by Boyle, Norman and Popham [8] as well as Claussen, Smits, Naess and Smith [9], they indicated that both upwardly and downwardly mobile people may carry with them a socialization from the class circumstances from which they depart. Nevertheless, previous research suggests that the longer people stay in their current class, the more they come to resemble this group [11]. Unfortunately, we had no information about the time people spent in the different class context, and the class weights in our study should therefore be interpreted with some caution. In spite of this shortcoming, however, the current study sheds light on the possibility that social mobility may impact on health through both acculturation and mobility effects.

### Methodological considerations

Compared to standard approaches, Diagonal Reference Models [45, 48] allowed us to analytically separate “being” in a certain class, from moving between them. Something which enabled us examine whether class transitions could have health implications, over and above the effects of prior and current class. However, although we have used the, to date, most appropriate method on prospective data shown to be representative of the same age cohort in Sweden overall [25], the study is subject to several limitations.

First, there are some factors which we have been unable to adjust for, for example any personal characteristics. Similarly, it has not been possible to capture and account for the reasons as to why people change class, whether the job relocation was by choice or involuntary, or took place within or between organizations. Consequently, we might be overlooking potential selection mechanisms that could act as a partial explanation.

Second, although our analyses give no such indication (neither self-rated health nor any measure of FSS seemed to affect the results), we cannot completely disregard the possibility of health selection. However, the likelihood that health status has been selecting people into different hierarchical occupations across their life course is fairly low both within our particular cohort [54] and in other contexts [55]. As a result, even though the interpretation of our results is limited by the fact that we measure current class concurrently with FSS at age 42, the risk of reverse causality seems low.

Third, since only two time points in mid-adulthood are used to operationalize mobility, people could have experienced some class transitions during the twelve years in between measurements that we have been unable to capture [56]. Most serious being the possibility that the people we define as immobile have in fact been mobile, but just ended up in the same class in 2007 and 1995. The extent of such a potential misclassification is, unfortunately, difficult to assess.

Fourth, the extent to which intragenerational social mobility is affected by time has, to date, received little theoretical and empirical attention. Whether the health implications of class transitions remain somewhat constant over the years or if they can be expected to decrease as people become more integrated in their new class is therefore unclear. Consequently, we do not know if, and in that case how our inability to account for the time spent in different classes affects the results, just that it is a limitation of the study. One the same note, the fairly high degree of mobility in our sample is a strength overall, but also suggests that people may not have reached “occupational maturity”. Our assessment of current class can therefore be seen only as a temporary endpoint to an overall mobility trajectory. Altogether, while our analytical approach offers new insights to the relationship between social mobility and health, it only provides a snapshot of and very limited insight as to the temporal variations of social mobility across the adult life course. Approaches such as ours should therefore be seen as complementary to strategies using more than two time points when modeling mobility.

Fifth and last, while we defined functional somatic symptoms as characterized by physical complaints in the absence somatic disease, we cannot ascertain that the measure is medically unexplained. Our operationalization is based on self-reported symptoms that have not been evaluated relative to a diagnosis. However, a recent systematic review and meta-analysis suggests that people with FSS rarely have a medical disorder to account for their symptoms [57]. In addition, while we acknowledge the overall discussion about the nature and classification of FSS [58] our assessment is similar to those used in population studies [59].

## Conclusion

By testing specific hypotheses derived from the dissociative, falling from grace, and acculturation theories, the present Swedish study examined whether intragenerational social mobility across 12 years in mid-adulthood could be linked to functional somatic symptoms. All in all, although our results provide limited support for any of the theories, the analyses indicate that upward movements in the class hierarchy could be potentially beneficial for stress-related health problems in mid-adulthood, while downward mobility seems to be of less importance for middle-age health complaints. The present study therefore adds new insight to the body of knowledge examining intragenerational social mobility on health, suggesting that while mobile people seem to be shaped by both their prior and current class context, perhaps there is also an effect of moving per se. As such, although our analytical approach needs to be complemented by similar studies performed in other contexts, future research examining mobility at more than two time points should be aware about the possibility that a detailed social mobility trajectory may include both acculturation processes and mobility effects.
